# Characterization of the Rac guanine nucleotide exchange factor P-Rex1 in platelets

**DOI:** 10.1186/1750-2187-6-11

**Published:** 2011-09-01

**Authors:** Joseph E Aslan, Alex M Spencer, Cassandra P Loren, Jiaqing Pang, Heidi C Welch, Daniel L Greenberg, Owen JT McCarty

**Affiliations:** 1 Department of Biomedical Engineering, Oregon Health & Science University, 3303 SW Bond Avenue, Mail Code CH13B, Portland, OR 97239 USA; 2 Department of Medicine, School of Medicine, Oregon Health & Science University, 3181 SW Sam Jackson Park Road, Portland, OR, 97239 USA; 3 Inositide Laboratory, The Babraham Institute, Babraham Research Campus, Cambridge, CB22 3AT, UK

**Keywords:** platelet signaling, cytoskeletal remodeling, GEF, small GTPase

## Abstract

**Background:**

Blood platelets undergo a carefully regulated change in shape to serve as the primary mediators of hemostasis and thrombosis. These processes manifest through platelet spreading and aggregation and are dependent on platelet actin cytoskeletal changes orchestrated by the Rho GTPase family member Rac1. To elucidate how Rac1 is regulated in platelets, we captured Rac1-interacting proteins from platelets and identified Rac1-associated proteins by mass spectrometry.

**Findings:**

Here, we demonstrate that Rac1 captures the Rac guanine nucleotide exchange factor P-Rex1 from platelet lysates. Western blotting experiments confirmed that P-Rex1 is expressed in platelets and associated with Rac1. To investigate the functional role of platelet P-Rex1, platelets from *P-Rex1^-/-^*-deficient mice were treated with platelet agonists or exposed to platelet activating surfaces of fibrinogen, collagen and thrombin. Platelets from *P-Rex1^-/- ^*mice responded to platelet agonists and activating surfaces similarly to wild type platelets.

**Conclusions:**

These findings suggest that P-Rex1 is not required for Rac1-mediated platelet activation and that the GEF activities of P-Rex1 may be more specific to GPCR chemokine receptor mediated processes in immune cells and tumor cells.

## Findings

Upon exposure to agonist signals of vascular injury, platelets spread out on sites of vessel damage to form thrombotic plugs [1,2]. During this process, platelets undergo an ordered series of shape changes that are determined by a spatial reorganization of the actin cytoskeleton [3]. These geometric changes that occur in the activated platelet are regulated by many of the same proteins that confer motility and regulate the cytoskeleton in nucleated cells, namely the Rho family of GTPases, including Cdc42, Rac1, and RhoA [4]. Accordingly, conditional knock-out mice models deficient in Rac1 do not undergo normal platelet spreading or aggregation and form a weak primary platelet plug over a site of vascular injury [5]. Similarly, constitutive deactivation of RhoA in platelets results in reduced platelet adhesion and an unstable thrombus [6].

Rho family GTPases are regulated in a cyclical manner by different classes of Rho-GTPase binding proteins. When platelets are stimulated to form a plug over the site of vascular injury, guanine nucleotide exchange factors (GEFs) such as Vav1 bind the Rac1 GTPase in its GDP conjugated form and catalyze a nucleotide exchange reaction to form Rac1-GTP [7]. Rac1-GTP is then able to bind downstream effecter proteins that regulate cytoskeletal proteins to form actin and myosin filaments. While Vav1 is known to control Rac1-based thrombotic activities in platelets, other well-established Rac1 GEFs have not been explored in regulating thrombosis.

**Figure 1:**
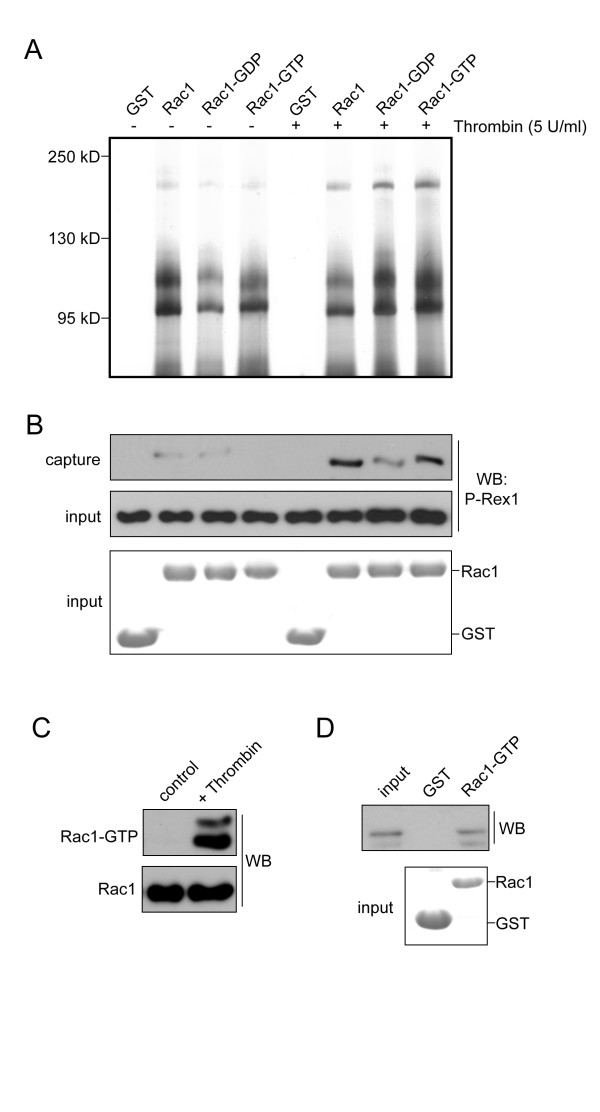
**Identification of P-Rex1 as a Rac1-associated protein in platelets**. Lysates from quiescent or thrombin stimulated platelets were incubated with glutathione agarose conjugated to GST-tagged Rac1, GDP-loaded Rac1, GTP-loaded Rac1 or GST alone. **(A) **Captured proteins were eluted into sample buffer and resolved by SDS-PAGE followed by silver staining. **(B) **Captured protein eluates and whole platelet lysates (input, middle panel) were probed for the presence of P-Rex1 by western blot (WB) with P-Rex1 antisera sc-85805 (Santa Cruz) as previously described [24,25]. Total Rac1-GST and GST protein inputs for capture experiments are shown by Coomassie stain. **(C) **Platelets from wild type mice (5 × 10^8^/ml) were treated with 1 U/ml of thrombin for 5 minutes and analyzed for Rac1 activation as previously described [5]. **(D) **Thrombin-stimulated mouse platelets were lysed in MPER buffer as previously described [19] and incubated with glutathione agarose conjugated to GTP-loaded Rac1-GST or GST alone for 1 hour at 4°C. Eluates were probed for mouse P-Rex1 capture by western blot (WB). Total P-Rex1 from mouse platelet lysates is shown as input (10% of total P-Rex1). Total Rac1-GST and GST protein inputs for capture experiments are shown by Coomassie stain.

To better understand how Rac1 is activated in platelets, we captured Rac1-associated proteins from platelet lysates and identified potential Rac1 regulatory proteins from thrombin-stimulated platelets by mass spectrometry. Platelets were purified from platelet rich plasma from healthy volunteers with Ficoll-Paque 400 [8] and adjusted to a concentration of 1 × 10^9^/ml. Lysates were prepared from resting platelets or platelets activated with 5 U/ml thrombin for 5 minutes. Immobilized Rac1-GST or GDP and GTP-loaded Rac1-GST were added to precleared lysates and incubated for 1 hour at 4°C. Rac1-associated proteins were eluted into Laemelli sample buffer and separated by PAGE. Silver-stained gel slabs from thrombin stimulated Rac1-GST eluates corresponding to 70 - 250 kD (Figure 1A, lanes 6, 7 and 8) were each separately digested with trypsin and resulting peptide fragments were analyzed with a ThermoFinnegan LTQ quadrupole linear ion trap spectophotometer fitted with an Ion Max nanospray source. Mass spectra were analyzed with Sequest software (Proteomics Shared Research Center, OHSU) and sequences were compared using Scaffold 2.1 software. Mass spectrometry capture experiments revealed that GTP-loaded Rac1-GST captured the Rac1 GEF P-Rex1 from thrombin-stimulated platelet lysates. Nine unique trypsin-digested P-Rex1 peptides were recovered (Table 1), representing 6% sequence coverage (103/1659 amino acids). Platelet lysates and Rac1-GST eluates were western blotted for the presence of P-Rex1 (Figure 1B), confirming that P-Rex1 is abundant in human platelets (input) and associated with Rac1 in vitro.

**Table 1:** Recovered P-Rex1 peptides

**Peptide (n)**	**Sequence**
1	EIDQDAYLQLFTK
2	LVDWLLAQGDCQTR
3	FLQSAFLHR
4	NQLLLALLK
5	GSLAEVAGLQVGR
6	TTDIPLEGYLLSPIQR
7	IACYQEFAAQLK
8	TTDIPLEGYLLSPIQR
9	LCVLNEIGTER

P-Rex1 functions as a specific Rac1 and Rac2 activator in neutrophils [9,10], endothelial cells [11] and breast cancer cells [12]. Intriguingly, the guanine nucleotide exchange activity of P-Rex1 is known to be regulated by both Gβ/γ and phosphoinositol-3,4,5 phosphate (PIP3) [9,13,14], suggesting that P-Rex1 could be involved in regulating G-protein coupled receptor (GPCR) pathways triggered by platelet agonists such as thrombin [15,16] and ADP [17]. Interestingly, we found that a ternary complex consisting of P-Rex1, Rac1-GTP and Gβ/γ occurs only in the thrombin-activated platelets (data not shown). P-Rex1 activity is also regulated through mTOR signaling [18], and recent work has described a role for mTOR in the activation of platelet Rac1 through an undetermined mechanism [19]. Accordingly, we hypothesized that P-Rex1 may function as an important Rac activator in response to stimulation of PARs and other platelet GPCRs.

**Figure 2:**
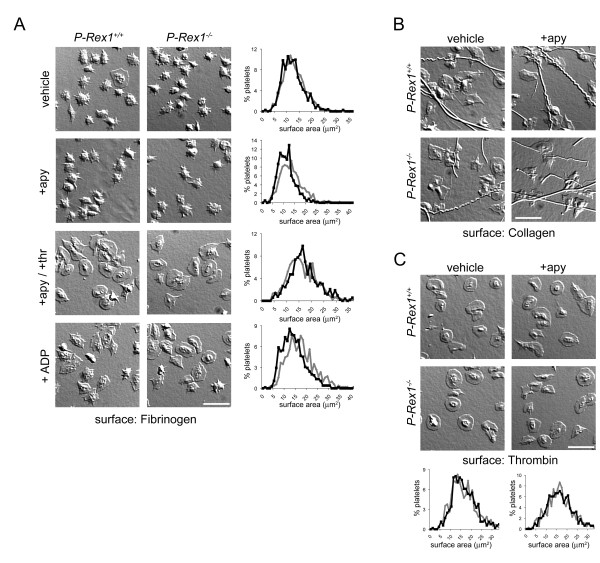
**Substrate surface spreading of *P-Rex1-/- *platelets**. Washed mouse platelets from wild type (*P-Rex1*^+/+^) or *P-Rex1*^-/- ^mice were placed on 100 μg/ml fibrinogen-coated **(A)**, 100 μg/ml fibrillar collagen-coated **(B) **or 50 μg/ml thrombin-coated **(C) **coverslips in the presence of vehicle, 2 U/ml apyrase (+apy), apyrase and 1 U/ml thrombin (+apy/+thr) or 10 μM ADP for 45 min at 37°C and imaged by DIC microscopy. The individual surface areas of 300 *P-Rex1*^+/+ ^(black line) and 300 *P-Rex1^-/- ^*(grey line) platelets were quantified using Image J software and plotted as a frequency distribution. DIC images and platelet surface area histograms are representative of > 3 experiments. Scale bar = 10 μm.

Thrombin markedly upregulated Rac1 activity in platelets from wild type mice as determined by capture of activated Rac1-GTP from quiescent versus stimulated platelet lysates [5] (Figure 1C). Protein capture and western blotting analyses confirmed that P-Rex1 is expressed in mouse platelets and capable of associating with GTP-loaded Rac1 (Figure 1D). To determine if P-Rex1 has a role in GPCR-triggered and Rac1-dependent platelet lamellipodia formation and surface spreading, we isolated platelets from *P-Rex1*-deficeint mice [10] and exposed them to platelet activating surfaces. Washed mouse platelets (2 × 10^7^/ml) from wild type (*P-Rex1^+/+^*) or *P-Rex1^-/- ^*mice were placed on 100 μg/ml fibrinogen-coated coverglass in the presence of vehicle, the ADP scavenger apyrase (2 U/ml), or the GPCR agonists thrombin (1 U/ml) or ADP (10 μM) for 45 minutes at 37°C and were examined using differential interference contrast (DIC) microscopy. Platelets from wild type and *P-Rex1^-/- ^*mice attached to fibrinogen surfaces at the same level (Figure 2A). The addition of the platelet GPCR agonists thrombin or ADP triggered platelet spreading on a surface of fibrinogen to a similar extent in both wild type and *P-Rex1^-/- ^*platelets (Figure 2A). Deletion of *P-Rex1 *similarly had no effect on the spreading of platelets on a surface of fibrillar collagen (Figure 2B) or thrombin (Figure 2C).

In conclusion, our study demonstrates that Rac1 interacts with P-Rex1 from platelets, however, the GEF activity of P-Rex1 is not likely essential to PAR and P2Y GPCR- and Rac1-mediated platelet lamellipodia formation and spreading. These results suggest that the activities of P-Rex1 may perhaps be more specific to GPCR chemokine receptor (CXCR)-mediated events in immune cells [10] and tumor cells [12,20-22]. While P-Rex1 alone does not appear to have a requisite role in activating Rac1 in platelets, recent studies suggest that P-Rex1 can work together with Vav1 to contribute to Rac1 activation [23]. Whether or not P-Rex1 has a secondary role in regulating platelet Rac1 activation and the potential context of such an accessorizing function of P-Rex1 in platelets remains to be determined.

## List of Abbreviations

ADP: adenosine 5'-diphosphate; CXCR: chemokine receptor; GEF: guanine nucleotide exchange factor; GST: glutathione S-transferase; GTP: guanosine-5'-triphosphate; GPCR: G-protein coupled receptor; MPER: Mammalian Protein Extraction Reagent; mTOR: mammalian target of rapamycin; PAR: protease activated receptor; PIP3: phosphoinositol-3,4,5 phosphate; P-Rex1: phosphatidylinositol-3,4,5-trisphosphate-dependent Rac exchange factor 1.

## Competing interests

The authors declare that they have no competing interests.

## Authors' contributions

All authors designed and carried out experiments. JEA wrote the manuscript. HCW supplied P*-Rex1^-/- ^*mice. All authors read and approved the final manuscript.

## References

[B1] Furie B Furie BC Thrombus formation in vivo J Clin Invest 2005 115 12 3355 3362 10.1172/JCI26987 16322780 PMC1297262

[B2] Ruggeri ZM Platelets in atherothrombosis Nat Med 2002 8 11 1227 1234 10.1038/nm1102-1227 12411949

[B3] Watson SP Platelet activation by extracellular matrix proteins in haemostasis and thrombosis Curr Pharm Des 2009 15 12 1358 1372 10.2174/138161209787846702 19355974

[B4] Bishop AL Hall A Rho GTPases and their effector proteins Biochem J 2000 348 Pt 2 241 255 10816416 PMC1221060

[B5] McCarty OJ Larson MK Auger JM Kalia N Atkinson BT Pearce AC Ruf S Henderson RB Tybulewicz VL Machesky LM Rac1 is essential for platelet lamellipodia formation and aggregate stability under flow J Biol Chem 2005 280 47 39474 39484 10.1074/jbc.M504672200 16195235 PMC1395485

[B6] Schoenwaelder SM Hughan SC Boniface K Fernando S Holdsworth M Thompson PE Salem HH Jackson SP RhoA sustains integrin alpha IIbbeta 3 adhesion contacts under high shear J Biol Chem 2002 277 17 14738 14746 11830597 10.1074/jbc.M200661200

[B7] Pearce AC Wilde JI Doody GM Best D Inoue O Vigorito E Tybulewicz VL Turner M Watson SP Vav1, but not Vav2, contributes to platelet aggregation by CRP and thrombin, but neither is required for regulation of phospholipase C Blood 2002 100 10 3561 3569 10.1182/blood.V100.10.3561 12411320

[B8] Andersen H Greenberg DL Fujikawa K Xu W Chung DW Davie EW Protease-activated receptor 1 is the primary mediator of thrombin-stimulated platelet procoagulant activity Proc Natl Acad Sci USA 1999 96 20 11189 11193 10.1073/pnas.96.20.11189 10500152 PMC18009

[B9] Welch HC Coadwell WJ Ellson CD Ferguson GJ Andrews SR Erdjument-Bromage H Tempst P Hawkins PT Stephens LR P-Rex1, a PtdIns(3,4,5)P3- and Gbetagamma-regulated guanine-nucleotide exchange factor for Rac Cell 2002 108 6 809 821 10.1016/S0092-8674(02)00663-3 11955434

[B10] Welch HC Condliffe AM Milne LJ Ferguson GJ Hill K Webb LM Okkenhaug K Coadwell WJ Andrews SR Thelen M P-Rex1 regulates neutrophil function Curr Biol 2005 15 20 1867 1873 10.1016/j.cub.2005.09.050 16243035

[B11] Carretero-Ortega J Walsh CT Hernandez-Garcia R Reyes-Cruz G Brown JH Vazquez-Prado J Phosphatidylinositol 3,4,5-triphosphate-dependent Rac exchanger 1 (P-Rex-1), a guanine nucleotide exchange factor for Rac, mediates angiogenic responses to stromal cell-derived factor-1/chemokine stromal cell derived factor-1 (SDF-1/CXCL-12) linked to Rac activation, endothelial cell migration, and in vitro angiogenesis Mol Pharmacol 2010 77 3 435 442 10.1124/mol.109.060400 20018810 PMC3202486

[B12] Sosa MS Lopez-Haber C Yang C Wang H Lemmon MA Busillo JM Luo J Benovic JL Klein-Szanto A Yagi H Identification of the Rac-GEF P-Rex1 as an essential mediator of ErbB signaling in breast cancer Mol Cell 2010 40 6 877 892 10.1016/j.molcel.2010.11.029 21172654 PMC3038344

[B13] Barber MA Donald S Thelen S Anderson KE Thelen M Welch HC Membrane translocation of P-Rex1 is mediated by G protein betagamma subunits and phosphoinositide 3-kinase J Biol Chem 2007 282 41 29967 29976 10.1074/jbc.M701877200 17698854

[B14] Hill K Krugmann S Andrews SR Coadwell WJ Finan P Welch HC Hawkins PT Stephens LR Regulation of P-Rex1 by phosphatidylinositol (3,4,5)-trisphosphate and Gbetagamma subunits J Biol Chem 2005 280 6 4166 4173 15545267 10.1074/jbc.M411262200

[B15] Huang JS Dong L Kozasa T Le Breton GC Signaling through G(alpha)13 switch region I is essential for protease-activated receptor 1-mediated human platelet shape change, aggregation, and secretion J Biol Chem 2007 282 14 10210 10222 10.1074/jbc.M605678200 17298951

[B16] Azim AC Barkalow K Chou J Hartwig JH Activation of the small GTPases, rac and cdc42, after ligation of the platelet PAR-1 receptor Blood 2000 95 3 959 964 10648409

[B17] Gachet C P2 receptors, platelet function and pharmacological implications Thromb Haemost 2008 99 3 466 472 18327393 10.1160/TH07-11-0673

[B18] Hernandez-Negrete I Carretero-Ortega J Rosenfeldt H Hernandez-Garcia R Calderon-Salinas JV Reyes-Cruz G Gutkind JS Vazquez-Prado J P-Rex1 links mammalian target of rapamycin signaling to Rac activation and cell migration J Biol Chem 2007 282 32 23708 23715 10.1074/jbc.M703771200 17565979

[B19] Aslan JE Tormoen GW Loren CP Pang J McCarty OJ S6K1 and mTOR regulate Rac1-driven platelet activation and aggregation Blood 2011 10.1182/blood-2011-02-331579 PMC3175787 21757621

[B20] Qin J Xie Y Wang B Hoshino M Wolff DW Zhao J Scofield MA Dowd FJ Lin MF Tu Y Upregulation of PIP3-dependent Rac exchanger 1 (P-Rex1) promotes prostate cancer metastasis Oncogene 2009 28 16 1853 1863 10.1038/onc.2009.30 19305425 PMC2672965

[B21] Johansson FK Goransson H Westermark B Expression analysis of genes involved in brain tumor progression driven by retroviral insertional mutagenesis in mice Oncogene 2005 24 24 3896 3905 10.1038/sj.onc.1208553 15750623

[B22] Montero JC Seoane S Ocana A Pandiella A P-Rex1 participates in Neuregulin-ErbB signal transduction and its expression correlates with patient outcome in breast cancer Oncogene 2011 30 9 1059 1071 10.1038/onc.2010.489 21042280

[B23] Lawson CD Donald S Anderson KE Patton DT Welch HC P-Rex1 and Vav1 cooperate in the regulation of formyl-methionyl-leucyl-phenylalanine-dependent neutrophil responses J Immunol 2011 186 3 1467 1476 10.4049/jimmunol.1002738 21178006

[B24] Aslan JE You H Williamson DM Endig J Youker RT Thomas L Shu H Du Y Milewski RL Brush MH Akt and 14-3-3 control a PACS-2 homeostatic switch that integrates membrane traffic with TRAIL-induced apoptosis Mol Cell 2009 34 4 497 509 10.1016/j.molcel.2009.04.011 19481529 PMC2744858

[B25] Itakura A Aslan JE Sinha S White-Adams TC Patel IA Meza-Romero R Vandenbark AA Burrows GG Offner H McCarty OJ Characterization of human platelet binding of recombinant T cell receptor ligand J Neuroinflammation 2010 7 75 10.1186/1742-2094-7-75 21059245 PMC2992052

